# Immigrant health access in Texas: policy, rhetoric, and fear in the Trump era

**DOI:** 10.1186/s12913-019-4167-1

**Published:** 2019-06-05

**Authors:** Timothy Callaghan, David J. Washburn, Katharine Nimmons, Delia Duchicela, Anoop Gurram, James Burdine

**Affiliations:** 1 Department of Health Policy and Management, Texas A&M School of Public Health, 212 Adriance Lab Rd., 1266 TAMU, College Station, TX 77843 USA; 2 Texas A&M College of Dentistry, 3302 Gaston Ave, Dallas, TX 75246 USA; 3 Office of Special Programs and Global Health, Texas A&M School of Public Health, 212 Adriance Lab Rd., 1266 TAMU, College Station, TX 77843 USA; 4 School of Public Health, Brown University, 121 S Main St, Providence, RI 02903 USA; 5 Department of Health Promotion and Community Health Sciences, Texas A&M School of Public Health, 212 Adriance Lab Rd., 1266 TAMU, College Station, TX 77843 USA

**Keywords:** Health access, Immigrant, President trump, Texas, Community health worker

## Abstract

**Background:**

Since the 2016 presidential election, reports have suggested that President Trump’s rhetoric and his administration’s proposed policies could be exacerbating barriers to accessing health care for undocumented as well as lawfully present immigrants and their families in the United States. However, very little empirical work has analyzed this possibility or detailed how these reports and rhetoric have altered the health seeking behavior of mixed immigration status families.

**Methods:**

Using a series of focus groups throughout Texas in both English and Spanish, this qualitative study analyzes changes to health access for immigrants. We consulted Community Health Workers to better understand the barriers encountered by their otherwise hard-to-reach undocumented clients and their families as they interface with the health system, revealing key insights about the changing nature of barriers to access under the Trump administration.

**Results:**

We identify four key themes about the changing nature of immigrant health access in the United States: growing fear of interacting with health and social services; that social networks are paradoxically limiting health access in the current political climate; that the administration’s rhetoric and proposed policies are impeding health seeking behavior; and that children are encountering new barriers to social program participation.

**Conclusions:**

The Trump administration, its proposed immigration policies, and his rhetoric are posing new and significant barriers to health access for immigrants and their families.

**Electronic supplementary material:**

The online version of this article (10.1186/s12913-019-4167-1) contains supplementary material, which is available to authorized users.

## Background

Since the 2016 presidential election, scholars have suggested that the Trump administration’s proposed immigration policies and rhetoric could exacerbate barriers to accessing social services and health care for vulnerable individuals in the United States [1–4]. A number of recent media reports highlight how anti-immigration policy rhetoric by the Trump administration has caused immigrants and their families to withdraw from participation in public assistance programs and to limit their interactions with public officials and health providers [5–8]. Hispanics, who make up approximately 18% of the population of the United States (nearly 59 million people) have faced a disproportionate share of these politically-driven barriers [9].

President Trump has emphasized immigration as a top priority through his rhetoric and his declaration of a national emergency to build a wall along the US-Mexico border. Additionally, news reports have emphasized proposed policy changes by the Trump administration to further restrict immigrant families (including lawfully-present non-citizens) from benefitting from a number of public programs including food subsidies and children’s health insurance [8, 10, 11]. This includes proposing that immigration officials consider immigrant use of government insurance and nutrition programs as a negative factor during legal residency applications [12–16].

These developments under the Trump administration may compound what scholars have recognized to be considerable barriers to health access for Hispanic populations in the United States. Even after the passage of the Affordable Care Act, which brought health coverage to 20 million Americans, a disproportionate share of Hispanics remain uninsured [17]. In 2016, Hispanics represented 33% of uninsured residents (and 18% of the total population) in the US while they made up much larger proportions of the uninsured in states like California (56%) and Texas (62%) with large Hispanic populations (39%) in each state [9, 18]. In both states, a large percentage of those born outside of the US arrive from Latin America, 50% in California and 69% in Texas, trends that have remained relatively consistent for decades [19].

The inequities and barriers found within the United States healthcare system are particularly problematic for immigrants. Permanent resident immigrants are only eligible for public health insurance programs like Medicaid and CHIP after five years in the United States, complicating health access for legal immigrants not covered by private, employer-sponsored insurance, which accounts for well over half of all insurance in the country [20–22]. Research has shown, including in examples in Canada and the United Kingdom, that barriers to health access are even more pronounced among *undocumented* immigrants and their children [23–25]. In the United States, undocumented immigrants are not eligible for any government run health insurance programs. Instead, they must rely on “a patchwork of Federally Qualified Health Centers, private charities, and hospital emergency departments” for needed health care services [26]. Notably, even as the resident children of undocumented immigrants are eligible for public health benefits through the Children’s Health Insurance Program, prior research demonstrates that these children have significantly fewer medical appointments and emergency room visits than the children of citizens, highlighting how the barriers faced by immigrant parents can affect their children [26]. While a lack of insurance presents an important barrier to accessing health care for Hispanic immigrants, prior research notes that access issues can be exacerbated by other barriers including the cost of care, transportation, the inability to take time away from work, child care, limited knowledge, language, gender, ethnicity, documentation status, and fear [27–31].

Recent perspectives from scholars studying Hispanic health suggests that these barriers could be intensifying under the Trump administration [1–4]. That said, to this point minimal empirical research has actually analyzed how access to health care services for immigrants has changed since President Trump’s election. This is particularly true for undocumented immigrants and their families who are expected to be most impacted by Trump’s policy efforts. Notably, research on undocumented immigrants and their families (mixed status families) can struggle to adequately capture the experiences of these families due to the difficulty of scholars gaining sufficient access to these vulnerable groups for analysis, especially in an era of growing distrust.

To overcome these challenges and provide timely evidence on how health access has changed for immigrants under the Trump administration, this empirical study relies on the experiences of Community Health Workers (CHWs) who work closely with immigrants and their families. As bridge figures who help to connect vulnerable populations with health and social services in a culturally appropriate manner, CHWs are uniquely positioned to provide critical insight into the challenges and barriers their clients face in accessing needed health care.

While CHWs have many different job titles and roles as health promotors, community health advocates, health navigators, and more, all CHWs are public health practitioners unified in their efforts to connect with the populations they serve, often through shared experience and cultural understanding [31–36]. Whether they work in the public, private, non-profit, or academic sectors, or just help as volunteers, their numbers are growing. In Texas, from 2008 to 2017, the number of certified CHWs grew to over 4000, a 600% increase over that time period, highlighting their growing influence in modern health care [37]. With Texas’ large Hispanic immigrant population, significant barriers to accessing needed care within that group, and the expanding role of CHWs as bridge figures in connecting marginalized individuals and families with services, this research provides important and timely insight into the changing nature of health access for a particularly vulnerable population in the Trump era.

## Methods

To investigate changes in immigrant health access, this research relies on a series of focus groups conducted with CHWs in Texas. Our study focuses on Texas due to its large undocumented Hispanic population as well as its growing population of CHWs. Participating CHWs were identified with the help of the National Community Health Worker Training Center. Participants took part in one of seven focus groups that occurred between 2017 and 2018 in Texas. The first two focus groups were conducted in the Brazos Valley - home to a large university in Texas - and included CHWs from 7 largely rural surrounding counties. The first of these focus groups was done in Spanish on October 21, 2017 and the second was carried out in English and Spanish on November 6, 2017. One focus group was conducted in Houston (December 9, 2017) and another in Dallas (March 23, 2018); both of which were carried out in English. Finally, we conducted three focus groups in border regions of the state. Two focus groups were carried out on February 10, 2018 in Spanish in McAllen, Texas. McAllen is near the eastern edge of the Texas-Mexico border and is the site of the Customs and Border Protection Processing Center, which has been central to news coverage of the Trump administration’s controversial family separation policy. Finally, one focus group was done on March 24, 2018 in Spanish in a colonia – an unincorporated border settlement – nearby El Paso and across from Cuidad de Juarez on the western Texas-Mexico border.

In total, our research included 66 CHWs working in varied environments across Texas including urban, rural, border, and non-border regions. All participants were state-certified CHWs and varied in levels of experience from just certified to over 20 years of experience. Our sample was 90.9% female, closely matching the 88% female CHW rate identified by the state of Texas [37]. Notably, our participants pointed to their diverse work environments in hospitals, clinics, non-profit organizations, and the community as well as their extensive experience working with immigrant clients in each of these settings.

Each focus group used for our analysis was two hours long and concentrated on CHW experiences with health access broadly. They included questions about the meaning of health access to CHWs, barriers to access for clients, ways for clients to overcome health barriers, programs clients participate in, and perceived changes in health access in the past few years. Critically, each question included follow-up probes designed to understand how experiences varied across clients with different backgrounds; most notably for Hispanics. These questions are available in Additional file 1. Focus groups were audiotaped, translated as needed, and then transcribed before analysis using the qualitative data software NVivo-11. For coding of the focus groups, we relied on a thematic analytic approach that was deductive - driven by existing research on health access and barriers to it - and inductive - allowing information that emerged from the transcribed materials to inform our results [38–43]. Qualitative data was coded independently by two researchers on the project and a high degree of similarity in coding across key themes was found between the investigators. All quotes included below are representative of other comments made by participants on our key themes.

## Results

Our discussions with CHWs throughout Texas revealed four key themes about the changing nature of health access for Hispanic immigrants and their families under the Trump administration: the pervasive role of fear as a barrier in current interactions with health and social services, how social networks can drive health seeking behavior in the current political environment, that President Trump, his rhetoric, and his administration’s proposed policies have impeded health access, and growing issues with health access for the children of immigrants under the Trump administration.

### Theme 1: growing fear of interacting with health and social services

Across focus groups, the most consistent finding was the pervasive way that fear is limiting access to needed health care and social services for mixed status families under the Trump administration. Concerns about deportation, particularly in an era of heightened Immigration and Customs Enforcement (ICE) interactions, are preventing many from seeking the care they need. As one CHW noted:

*“The people who do not have health insurance or legal status here, they fear to go to the clinic. I have known people who are dying at home, they prefer to endure a tooth ache, a stomach ache, than to be seen by a doctor. They say ‘if they catch me and know my name, they will take me out of the country’. (Brazos Valley)”*

While these fears are ever-present in daily life for undocumented immigrants, our participants noted growing fear in the current political environment. For example, one CHW in McAllen noted that “when he (President Trump) became president, it was noticeable the fear was rising.” Another CHW in El Paso stated that “fear has become a factor of life.” It is “immobilizing people” and they “are not even going out” even for work on days when immigration checkpoints are in place.

Interestingly, CHWs also pointed out how this fear can become particularly problematic during public health emergencies like Hurricane Harvey. With up to 60 in. of rain resulting in massive flooding, the city of Houston, the state of Texas, and the federal government marshalled all relevant emergency resources in the wake of the hurricane. This included the use of 35 Customs and Border Patrol boats for water rescues [44]. CHWs in Houston however discussed at length how the use of Customs and Border Patrol boats for water rescues scared away undocumented immigrants and their families in need of rescue. CHWs noted that many families chose to brave potentially life threatening and unhealthy floodwaters rather than be rescued and risk the possibility of deportation, even as the news reported that border patrol agents would not be enforcing immigration laws [44].

Critically, our participants acknowledged that while CHWs are uniquely positioned to work against this fear given their role as bridge figures to vulnerable populations, they too are confronted with this barrier as health providers due to their status as authority figures. CHWs for example discussed how “Unfortunately, they [community members] are afraid of us [CHWs]…We go through the *colonias*, and people say to leave the information in the mailbox or the window. They hardly open the door because they are afraid. (McAllen)”.

### Theme 2: social networks identify patterns that may not exist

During discussions of the omnipresent fear of deportation under President Trump, CHWs also pointed to how that fear in their clients can be amplified by the personal experiences of other community members. Participants noted how being contacted by the authorities regarding immigration status in the days and weeks after medical appointments would sometimes be blamed on health service providers, an idea which then permeates throughout the community by word-of-mouth in the current political climate. This idea was best articulated by one CHW who described the issue by saying:

*“Locally... it’s like ‘no vayas’... don't go to that clinic because that receptionist is known to call ICE on you or is going to report you. I went to XYZ clinic and then I got a phone call or then I got [a] visit at my house or something. Because these are all families that are at various stages of their immigration process, either they have filed something or they're waiting to hear back from ICE. And then all of a sudden they get a letter in the mail, stating that it is an order of deportation or… removal. They start making these connections that well maybe it's because I'm undocumented, because I speak Spanish… there's a hyper-vigilance that occurs in our communities, so those are the rumors. [They] are very damaging and it’s another barrier to accessing health care. (Dallas)”*

Through social contacts and word-of-mouth exchanges, undocumented individuals and their family members are learning to avoid key venues for health services, further limiting access to care.

### Theme 3: changes in executive rhetoric and enforcement are impeding access to health

While fear of deportation and the sharing of (mis)information have long been barriers to health access for undocumented immigrants and their families [45–47], all seven of our focus groups highlighted the fact that the election of President Trump, his rhetoric, and his administration’s proposed policies have complicated matters and decreased health access. Most notably, across focus groups our CHWs pointed out that the rhetoric of the President has important downstream consequences for undocumented Hispanics accessing care. This idea was best exemplified by a CHW working at a health clinic that serves undocumented immigrants:

*“It was like a Friday… he [Trump] was threatening to deport people left and right. That next Monday and almost the entire week, our clinics, everybody didn't even cancel, they didn't show up. They were terrified. (Houston)”*

As important as President Trump’s rhetoric is, efforts at immigration enforcement have been just as influential according to our focus groups, particularly as it relates to limiting transportation:

*“The other thing to understand under a Trump Administration… the border is no longer the border that we used to know. The border is now throughout Texas. So the checkpoints are throughout Texas and residents will avoid where checkpoints are or will avoid driving all together because they don't have a driver's license. (Dallas)”*

While CHWs reported that many of their clients faced transportation barriers to accessing care, this obstacle has become more of a challenge as checkpoints are perceived to be more frequent and showing up farther away from border areas. Fearful of being stopped while travelling to clinics, some immigrants are no longer seeking out the care they need. For example, one CHW stated that the “news says the border patrol has increased in number. [People] are afraid to leave from their homes and go to a clinic. (McAllen)”.

### Theme 4: children are more at risk

According to our participants, the policy discussions of the Trump administration have been particularly impactful for children. In addition to controversial administration policies that have seen family separation at immigration centers and debates over the Deferred Action for Childhood Arrivals (DACA) program [48, 49], children of immigrant parents have faced significant difficulties in accessing needed nutrition in the past two years. In particular, CHWs in multiple focus groups discussed how the administration’s proposed policy changes have led to unintended consequences with the Supplemental Nutrition Assistance Program (SNAP) and Women, Infants and Children (WIC) Food and Nutrition Services for their clients:

*“if you are receiving benefits and you're undocumented they were going to deport you... he [Trump] was working on getting access to immigration status for people who are receiving public assistance, which is SNAP not Medicaid. So a lot of people did let SNAP benefits expire and could not feed their children...it was because of the fear that their information would be given to ICE and they would be deported. (Dallas)”*

This CHW reported widespread fear that the Trump administration might use the documentation status of current and legally-eligible recipients of public benefits in future deportation efforts, which has important consequences for child nutrition. Another CHW who works to help clients apply for state benefits in Dallas echoed these sentiments by saying that many clients “say I’m not renewing because if I renew I’m going to get deported. So we lost a lot of people… their kids are the ones that were suffering….” This was further echoed in McAllen, where a third CHW stated that growing fear has resulted in declining WIC participation, noting that children “deserve help [but] they do not go; it’s scary.”

## Discussion

In our analysis of immigrant experiences with health access, we have identified several noteworthy patterns. First, as reported by CHWs in our focus groups, fear remains a pervasive and problematic barrier for undocumented immigrants and their families attempting to access care. Many undocumented individuals and their families are choosing to forego obtaining needed health services due to the potential threat of their deportation or the deportation of a loved one. As such, these individuals do not get preventative care or seek out services for emergent conditions. Just as important, CHWs in our sample have suggested that in the current heightened climate of fear, even when immigrants do seek out care, they are learning to avoid certain clinics through word-of-mouth. CHWs have suggested that immigrants and their families are (perhaps erroneously) connecting notifications regarding immigration status to their choice to seek health services, creating a cycle of misinformation connecting health seeking behavior to immigration notifications.

Critically, our focus groups highlighted that CHWs perceive that access to health care for immigrants and their families has declined under the Trump administration. The President’s focus on immigration as a top policy priority has resulted in perceived increases in immigration enforcement throughout the state, decreasing the likelihood that a cautious and vulnerable population will seek out needed care. Perhaps more importantly for a President noted for his rhetoric through Twitter and other outlets, families appear to hear and react to the President’s statements. With CHWs in our sample pointing to missed appointments by patients in the wake of presidential rhetoric on immigration, it appears that the bully pulpit may now be a barrier to immigrant health access.

Importantly, our results suggest that this rhetoric has been particularly problematic for children’s nutrition. CHWs in our sample noted declines in SNAP and WIC participation by families of immigrants during the Trump administration. Our findings match recent quantitative research by Allison Bovell-Ammon et al. 2018, which suggests that SNAP participation by eligible immigrant families who have been in the US less than five years “dropped by nearly 10 percent in the first half of 2018” [14]. With no policy changes occurring during our period of analysis, this decline in nutrition assistance is likely connected to President Trump’s rhetoric on immigration. It is also possible that potential changes to the “public charge” rule in the US being debated during our period of analysis could have contributed to this decline. Specifically, proposed public charge rule changes that would have made it harder for immigrants to obtain green cards or to extend temporary visas if they or their dependents participated in these programs could have led some immigrant families to leave these programs pre-emptively to avoid losing their opportunities for future legal status [15, 16].

While the primary purpose of our research was to identify changes in health access for immigrants and their families in the Trump era, our findings also point to potential future actions that could be taken to limit barriers to access. First, our findings suggest that more work needs to be done to mitigate the fears of this vulnerable group so that they still seek out needed health services. Future research should explore strategies to increase trust in the health system and to disassociate health seeking from generalized immigration fear. We suspect that CHWs may be uniquely positioned to serve this purpose given their primary role as bridge figures – providing information, correcting misinformation, ensuring children get adequate nutrition, and identifying trustworthy health providers. That said, research is needed to understand effective strategies for CHWs to build that trust and if additional training of CHWs in this area is warranted. Beyond building on existing CHW capacities to improve trust and reduce fear, our findings illustrate the power of rhetoric and the need for political actors to employ their bully pulpits carefully. Future research should explore the types of political messages that induce fear in vulnerable groups so that we can better understand the rhetorical triggers of fear.

Despite the importance of these findings to our understanding of Hispanic health access in the Trump era, there are limitations to this study that are worth noting. First, it is necessary to acknowledge we did not speak with immigrants themselves in our study. We do not have first-hand accounts of immigrants’ health seeking behavior and as such, our findings might miss some of the nuance that only first-hand accounts can provide. Relatedly, the choice to rely on CHWs as informants could be argued to contribute towards the silencing of an already vulnerable and marginalized group of immigrants and their families.

That said, we made the choice to focus on CHWs because we felt that the ethical and methodological barriers to research with mixed status families instead of CHWs was too high. Methodologically, we were concerned that we would not be able to identify a sufficiently large sample of respondents, particularly with mixed documentation statuses, to allow for reasonable conclusions to be drawn from any findings. Ethically, we were concerned about any perceived risks undocumented immigrants may feel about participating in the project, particularly when discussing controversial topics like Trump administration policies.

We feel that CHWs serve as a useful proxy given their unique role as bridge figures to vulnerable groups like the undocumented. They are able to provide important insight into their experiences with this vulnerable group as they interact with our health system without the same worry that many vulnerable immigrants would have about participating in academic research. Notably however, by focusing on CHWs our results inherently fail to capture the experiences of immigrants and their families who do not intersect with these bridge figures. Thus, as troubling as many of our findings appear, they may in fact underestimate the realities of those who are more disenfranchised.

Second, by focusing our research on Texas alone, our analysis might miss information that could be gained by studying more states. Additional qualitative research on immigrant health access in states with large Hispanic immigrant populations like California and Arizona could provide important detail about the generalizability of our findings outside of Texas. Relatedly, qualitative research on immigrant families in non-border states could lead to insights about any differences in health access between border and non-border regions of the US.

Next, by relying on qualitative focus groups for this research, our study is not broadly representative of immigrant experiences and might therefore miss trends that quantitative analysis could identify. Focus group research makes it difficult to know if CHW statements are based on first-hand experiences, stories they have heard from other health workers, or just rumors. As such, future research should quantitatively explore the trends identified here. Given the professional nature of our sample and with our participants’ comments about declining SNAP participation already being verified with quantitative research, we do have a high degree of confidence in our findings. That said, quantitative research, with a particular emphasis on verifying our findings would be valuable. We believe that survey-based research designs studying CHW experiences with their clients could also be fruitful to future research in this area.

## Conclusions

Ultimately, despite these concerns, our research represents an important step forward in our understanding of changes in Hispanic health care access in Texas, especially for mixed status families and those who fear deportation in the Trump era. We have shown the pervasive role of fear as a barrier, how negative experiences spread by word-of-mouth through communities, and most notably, suggested that President Trump, his rhetoric, and his administration’s proposed policies could be threatening access to care for this vulnerable population.

## Additional file

Additional file 1: Focus Group Questions. Additional file 1 provides the list of questions asked to Community Health Workers as part of focus groups held throughout the state of Texas. These questions are provided in English and Spanish. (DOCX 18 kb)

## Data Availability

The transcripts of the focus groups used for the analysis presented here are available upon request. To protect the privacy of participants, no names are used in our manuscript here.

## Abbreviations

CHW: Community health worker
DACA: Deferred action for childhood arrivals program
ICE: U.S. Immigration and customs enforcement
SNAP: Supplemental nutrition assistance program
WIC: Special supplemental nutrition program for women, infants, and children
