# Supplementary material for: Immigrant health access in Texas: policy, rhetoric, and fear in the Trump era
Source: BMC Health Serv Res. 2019 Jun 5;19:342. doi: 10.1186/s12913-019-4167-1 (PMC6549327; doi:10.1186/s12913-019-4167-1)
Supplement: Supplementary file 1 — Focus Group Questions. Additional file 1 provides the list of questions asked to Community Health Workers as part of focus groups held throughout the state of Texas. These questions are provided in English and Spanish. (DOCX 18 kb) [file 12913_2019_4167_MOESM1_ESM.docx]

**Additional File – Focus Group Questions**

Preguntas del Grupo de Enfoque Promotores/as de Salud

Questions for Focus Group of Health Promoters

1. Nosotros sabemos que Promotores/as de Salud trabajan en una variedad de lugares en la comunidad, hospitales, etc. haciendo una variedad de actividades. ¿Puede usted decirnos sobre el trabajo que usted hace como un/a Promotor/a de Salud?

We know that health promoters work in a variety of settings in the community, hospitals, etc. doing a variety of things. Could you tell us about the work that you do as a health promoter?

- Posibles preguntas de seguimiento:

Possible follow-up questions:

- - ¿Con qué poblaciones usted trabaja?

What populations do you work with?

1. ¿Qué significa el termino ‘acceso a la salud’ para usted personalmente?

What does the term access to care mean to you personally?

- Posibles preguntas de seguimiento:

Possible follow up questions:

- - ¿Como un/a Promotor/a de Salud?

As a health promoter?

- - ¿Para sus clientes?

For your clients?

- - ¿Es diferente para personas de distintos grupos sociales o étnicos? Latinos…

Is it different for people with different backgrounds? Latinos…

- - ¿Qué tipos de barreras existen en el acceso a la salud?

What type of barriers exist in access to health care?

1. Hemos identificado un numero de barreras para el acceso a la salud. ¿Podrian nombrar otras barreras importantes?

We have identified a number of barriers to access to care. Could you name any other important barriers?

- Posibles preguntas de seguimiento:

Possible follow up questions:

- - ¿En su opinion, cuales son las más importantes?

In your opinion, which of these is the most important?

¿Cuáles de estas barreras son más difíciles para personas de distintos grupos sociales o étnicos? Latinos…

Which of these barriers are most difficult for people with different backgrounds? Latinos…

- - ¿Para sus clientes, es importante cuánto tiempo han vivido en la comunidad o el país? ¿Cómo?

For the people you work with, does the amount of time that they have been in the community/country matter? How?

1. Pensando en lo que hemos discutido de estas barreras, algunos lo han superado y otros no. ¿Por qué se hace más fácil para ciertas personas?

Given what we have discussed about these barriers, some people overcome them and some people don’t. Why is it easier for some people to overcome them?

- Posibles preguntas de seguimiento:

Possible follow up questions:

- - ¿Es diferente para personas de distintos grupos sociales o étnicos? Latinos…

Is it different for the certain groups of people with different backgrounds? Latinos…

1. ¿En qué programas y servicios de salud participan sus clientes?

Which programs and health services do your clients participate in?

1. ¿Usted encuentra que la gente está dudosa de participar en programas de salud dirigidos por el gobierno?

Do you find that people that you work with are hesitant to participate in health programs run by the government?

- Posibles preguntas de seguimiento:

Possible follow up questions:

- - ¿Es diferente para personas de distintos grupos sociales o étnicos? Latinos…

Is it different for the certain groups of people with different backgrounds? Latinos…

- - ¿Para cuáles programas se ve la gente mas dudosa de participar? ¿Y por qué?)

Are there programs where you see people more hesitant to participate? Why?

1. Dada su experiencia unica, ¿qué piensa usted que debería hacerse para mejorar el acceso a los servicios de salud para sus clientes y la gente en general?

Given your unique experience, what do you think should be done to make access to health care services easier for your clients and people in general?

1. ¿Qué lo haría más fácil para que los promotores pudieran mejorar aceso a la salud? ¿Para Latinos?

What would make it easier for health promoters to further improve health access? For Latinos?
